# Heterogeneity in clinical prognosis, immune infiltration and molecular characteristics of three glycolytic subtypes in lower-grade gliomas

**DOI:** 10.3389/fonc.2023.1180662

**Published:** 2023-05-18

**Authors:** Shenglian Wu, Lulu Sheng, Shucai Fan, Xi Guo, Biao Zhu, Cheng Wu, Bing Lei

**Affiliations:** Department of Neurosurgery, Cancer Center, Zhejiang Provincial People’s Hospital Affiliated to Hangzhou Medical College, Hangzhou, ZheJiang, China

**Keywords:** glycolysis, metabolism, stemness, immunity, lower-grade glioma

## Abstract

**Background and purpose:**

Lower-grade gliomas (LGG) exhibit a wide range of metabolic pathway changes, and metabolic reprogramming can be largely seen as a result of oncogenic driving events. Glycolysis, an important pathway of tumor energy source, has been poorly studied in gliomas. The aim of this article is to analyze the relationship between glycolysis and lower-grade glioma development and prognosis in order to explore the heterogeneous relevance of glycolysis in lower-grade gliomas.

**Methods and results:**

Our study searched the TCGA database and identified three glycolytic subtypes with significant prognostic differences by unsupervised clustering analysis of core glycolytic genes, named C1, C2, and C3. By analysis of clinical prognosis, somatic cell variation, and immune infiltration, we found that C3 had the best prognosis with molecular features of IDHmut-codel, followed by C1 with major molecular features of IDHmut-non-codel, G -CIMP high subtype, while C2 had the worst prognosis, mainly exhibiting IDHwt, G-CIMP low and mesenchymal-like subtypes with seven important CNV features, including CDKN2A/B deletion, chr7 gain and chr10 deletion, chr19/20 co-gain, EGFR amplification and PDGFRA/B deletion phenotypes were significantly increased, with the highest level of stemness and significant T-cell depletion features. Finally, to quantify the level of abnormal glycolysis and its impact on prognosis, we developed GlySig to reflect the glycolytic activity of LGG and integrated molecular features to construct nomogram that can be independently assessed to predict prognosis.

**Conclusions:**

Our study analyzed the tumor characteristics of different glycolytic states, and our findings explain and describe the heterogeneity of glycolytic metabolism within diffuse LGGs.

## 1 Introduction

Diffuse lower-grade gliomas are defined as WHO grade 2 and WHO grade 3 gliomas (hereafter referred to as lower-grade gliomas) (1), with a median survival of 78.1 months for WHO grade 2 gliomas and 37.6 months for WHO grade 3 gliomas (2). For many years, the prognosis of lower-grade gliomas has not been significantly improved after standardized treatment using surgery with chemo-radiotherapy, posing a major challenge to human health. In recent years, immunotherapy has brought light to the treatment of tumors, but there is no definite efficacy in glioma, which has been shown to be closely related to the cold tumor phenotype mediated by the immunosuppressive microenvironment (3), so an intensive study of the immunosuppressive micro-environment is the main way to achieve a major breakthrough in the treatment of glioma.

Tumor glycolysis is the phenomenon whereby cancer cells tend to generate energy through glycolysis rather than mitochondrial oxidative phosphorylation even in the presence of sufficient oxygen, known as the Warburg effect (4). Enhanced glycolysis rapidly produces large amounts of ATP and also promotes the synthesis of macromolecules (nucleotides, fatty acids, proteins) required for tumor proliferation (5). In addition, tumor cells have a direct immunosuppressive effect on the tumor microenvironment through competition with effector T cells for glucose uptake as well as hypoxia and lactate accumulation due to rapid glucose utilization (6–8). Studies have shown that increased glycolysis promotes an aggressive phenotype of gliomas (9, 10). Therefore, targeting glycolysis has become a therapeutic direction for glioma, but glioma is a complex tumor microenvironment with metabolic differences between different cell subpopulations of the same glioma (11), and the potential metabolic differences between glioma subtypes remain unclear (12). Therefore, further understanding of the metabolic differences between different gliomas is crucial for individualized treatment of gliomas.

In this article, we identified five sets of genes associated with glycolytic processes enriched in LGG from MSiDB data, performed unsupervised clustering analysis based on 26 of these differential genes, classified glycolytic types in lower-grade gliomas into three subtypes, and analytically described clinical prognosis, genetic variation, immune infiltration, and tumor phenotype for the three subtypes, and used core prognostic genes. A lasso risk model was developed for the prognosis of lower-grade gliomas. Our findings demonstrated the metabolic heterogeneity of lower-grade gliomas and describe the clinical significance, molecular differences and immunological features of three different glycolytic subtypes, providing new targets for personalized treatment of glioma.

## 2 Material and methods

### 2.1 Collection and preprocessing of data

The RNA-seq matrices, copy number variation (CNV) profile, clinical data, and somatic mutation profiles (Varscan) for all LGG samples were downloaded from the TCGA database (https://portal.gdc.cancer.gov/). The gene sets for evaluating the glycolytic metabolic processes were downloaded from the MSigDB database of the GSEA (http://www.gsea-msigdb.org/gsea/index.jsp). The supervised DNA methylation clusters, molecular subtypes, and immunophenotype profiles of TCGA samples were obtained from the supplementary material of previous studies (1, 13, 14). Also, we have chosen three cohorts, including mRNA-array_301, mRNAseq_325, and mRNAseq_693 from the CGGA database (http://www.cgga.org.cn/). Normal samples were obtained from the GTEx database (https://gtexportal.org/).

Next, we consolidated data from the same sources. A total of 509 TCGA samples simultaneously have clinical data, RNA-seq information, and mutational information. As for the CGGA cohort, 592 LGG samples had both gene expression and overall survival (OS) information. In the GTEx cohort, 207 normal samples were of cortical origin.

### 2.2 Methods for identifying glycolysis-related subtypes

Firstly, we evaluated the differences in the enrichment of 17 glycolysis-related pathways between normal cortical samples and the TCGA LGG cohort with the GSEA software (version 4.3.2). After 1000 iterations, a total of 5 pathways were found to be highly enriched in the TCGA LGG cohort. Next, the expression of the genes contained in the five signaling pathways was extracted, and the “edgeR” method was used to find differentially expressed genes (DEGs). A total of 26 DEGs were identified as key genes. Glycolysis-related subtypes were identified based on the consistent clustering method and the expression matrices of these 26 DEGs. Consistent clustering analysis was implemented based on the “clusterProfiler” package.

### 2.3 ssGSEA and evaluation of the immune microenvironment

The ssGSEA allows the assignment of enrichment levels for selected gene sets in each sample within the given data sets. Our study involves multiple genes sets about immune functions, metabolism, epithelial-mesenchymal transition (EMT) process, extracellular matrix (ECM), and cancer signaling pathways. These gene sets were obtained from public databases or published works (http://matrisome.org/. https://www.immport.org/. https://www.rndsystems.com/) (15–17). With the “GSVA” package, we calculated the ssGSEA score levels for these gene sets in LGG samples.

As for the assignment of immune cell infiltration, The CIBERSORT algorithm was used to forecast the infiltration of 22 kinds of immune cells. The main package was named “CIBERSORT”, and it was obtained from GitHub (https://github.com/) (18). ESTIMATE algorithm is based on the “ estimate “ package for evaluating the condition of the immune microenvironment (19). In addition, we utilized the ImmuCellAI algorithm to assess the lymphocyte infiltration levels (http://bioinfo.life.hust.edu.cn/web/Imm-uCellAI/) (20). The TIDE algorithm was used to assess the T-cell exhaustion levels (http://tide.dfci.harvard.edu./) (21). The TIP algorithm was used to evaluate the immune cycle in LGG samples (22).

### 2.4 Genetic variation analysis

This part is mainly based on the “maftools” package (23). Waterfall plots were plotted with the “oncoplot” function. The lollipop plots were painted with the “lollipopPlot” function. Correlations of 30 top mutated genes were obtained by Fisher’s exact test with the CoMEt algorithm provided by the “somaticInteractions” function. The enrichment levels and activities of cancer signaling pathways were visualized by the “OncogenicPathways” function.

### 2.5 GlySig construction with LASSO regression

Based on 26 key genes, OS time, and OS status, GlySig was constructed with the least absolute shrinkage and selection operator (LASSO) regression. The “createDataPartition” function in the “caret” package equally and randomly divided the TCGA LGG cohort into the train and test sets. After being examined by the chi-square analysis, there was no difference in the distribution of clinical traits between the train and test sets. Next, based on the train set, the 10-fold cross-validation was used to determine the best penalty coefficient (log (λ) = -3.3). With this λ value and 11 of 26 key genes had nonzero Coef value. After being validated by univariate Cox analysis, all these genes retained prognostic significance. Therefore, these 11 key genes were determined as lasso genes, and the GlySig was constructed according to the following equation: ). In the CGGA cohort, we calculated the GlySig with the identical formula and Coef value.

### 2.6 Construction of nomogram and DCA analysis

The construction of the nomogram was based on Cox regression and relied on the “rms” and “regplot” packages. Finally, age, GlySig, and grade were identified as independent prognostic factors, and nomograms were constructed on this basis. The comparison of net clinical benefit was based on decision curve analysis (DCA). Operation and visualization of DCA were based on the “ggDCA” package.

### 2.7 Functional enrichment analysis

The “org.Hs.eg.db” package could convert the gene symbols into ensemble IDs, which could be identified by the algorithm. The “clusterProfiler” package was used to perform the Gene Ontology (GO) and the Kyoto Protocol Encyclopedia of Genes and Genomes (KEGG) functional enrichment analyses (24). Only GO and KEGG pathways with p<0.05 were retained. The visualization of these results was implemented based on the “enrichplot” and “ggplot2” packages.

### 2.8 Statistical methods, algorithms, and software

The Kruskal-Wallis test was used to compare differences between subtypes or subgroups. Co-expression analysis was performed with the Spearman correlation analysis. The Kaplan-Meier (K-M) method and the log-rank test were used for evaluating prognostic differences. The univariate Cox analysis was used to detect whether the variables were risk factors for prognosis, while the multivariate Cox analysis was used to determine whether the variables were independent prognostic biomarkers. The accuracy of prognostic prediction was examined with ROC curves, and the area under the curves (AUC) reflects the accuracy of biomarkers. The identification of DEGs was based on the “edgeR” package.

This study was performed mainly based on R version 4.0.4. The “pheatmap” package was used for plotting heatmaps. “ggplot2”, “ggpubr”, “ggExtra”, “plyr”, and “reshape2” packages could be used for plotting multiple figures, such as box plots, bar plots, and scatter diagrams. Forest plots were painted with the “forestplot” package, and ROC curves were plotted by the “timeROC” program. Principal component analysis (PCA) was implemented with the “ limma” package and could be visualized with the “ggplot2” package. K-M curves were plotted by the “survival” and “survminer” packages. In addition, some Perl scripts participated in the pre-processing of data (Strawberry-Perl-5.32.1.1).

## 3 Results

### 3.1 Identification of glycolysis-related subtypes

**Figure 1:**
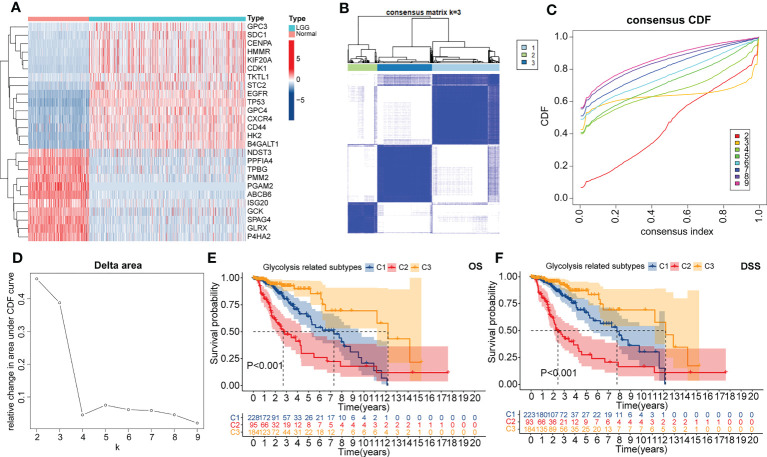
The heatmap presented the differential expressed genes (DEGs) between normal cortical samples in the GTEx cohort and LGG samples in the TCGA cohort **(A)**. Consensus heatmap of clustering similarity between three glycolysis-related subtypes **(B)**. Correlation of the CDF value with the consensus index. In this figure, different colors represented the varied k (cluster number) value **(C)**. Correlation of k value with relative changes in the area under the CDF value **(D)**. Overall survival (OS) **(E)** and disease-specific survival (DSS) **(F)** dependent K-M curves between three glycolysis-related subtypes.

To better understand the mechanism involved in the abnormal glycolytic metabolism in LGG samples, we collected 17 glycolysis-related alternative gene sets from the MSigDB database in the GSEA platform. By comparing with the normal cortical cohort, we found that five of the 17 alternative gene sets (including GOBP_FRUCTOSE_1_6_BISPHOSPHATE_METABOLIC_PROCESS; HALLMARK_GLYCOLYSIS; REACTOME_GLYCOLYSIS; REGULATION_OF_GLYCOLYSIS_BY_FRUCTOSE_2_6_BISPHOSPHATE_METABOLISM; and GLYCOLYSIS_IN_SENESCENCE) were highly enriched in the TCGA LGG cohort (**Supplementary Figures S1A–E**). These five selected gene sets contained a total of non-duplicated 231 genes, 26 of which were significantly differentially expressed between normal cortical and LGG samples (|logFC|>1, FDR<0.05, **Figure 1A**). Therefore, these 26 DEGs were identified as key genes of the glycolysis process in LGG samples.

Next, glycolysis-related subtypes were identified with the consistent clustering method and the expression of 26 key genes. The most significant change in the CDF value was observed when the number of clusters was three. Also, the mutual interference between different clusters is minimized in this condition (**Figures 1A–D**). Therefore, the TCGA LGG cohort was finally clustered into three subtypes, named C1, C2, and C3, respectively. Encouragingly, significant differences in principal components were observed between these three subtypes (**Supplementary Figures S1F**). Also, both OS and DSS-dependent K-M curves suggested that C3 had the best prognosis, followed by C1, while the worst prognosis was observed for C2 (**Figures 1E, F**). Therefore, our clustering strategy was reliable, and the glycolysis-related subtypes have the potential to be applied in the prognostic stratification of LGG.

### 3.2 Correlation of molecular profiles with glycolysis-related subtypes

**Figure 2:**
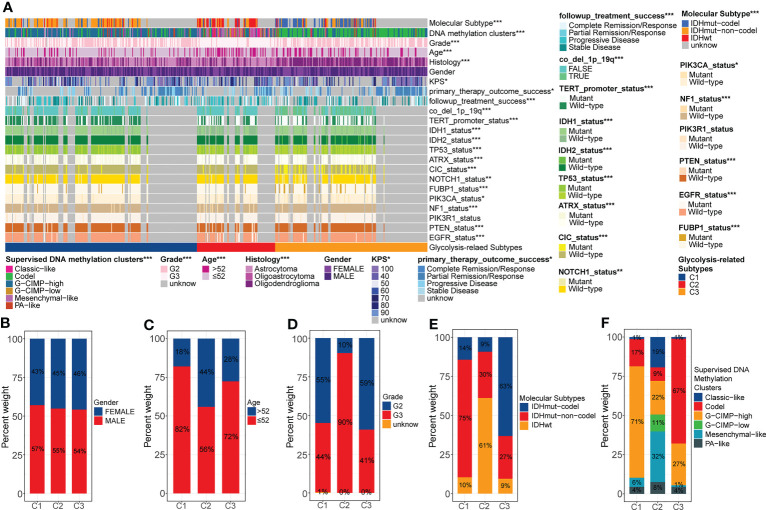
Overall review of the differential distribution of molecular profiles between three glycolysis-related subtypes **(A)**. Differential distribution of gender **(B)**, age **(C)**, grade **(D)**, molecular subtypes **(E)**, and supervised DNA methylation clusters **(F)** between three glycolysis-related subtypes. p<0.05 was presented with “*”, p<0.01 was presented with “**”, p<0.001was presented with “***”.

We noted that there was extensive molecular profile heterogeneity among these three glycolysis-related subtypes, which might be related to the variation in prognosis (**Figure 2A**). In terms of clinical traits, gender was evenly distributed among these three subtypes (**Figure 2B**). However, 44% of C2 patients were senior (>52 years), much higher than the 18% in C1 and 28% in C3 (**Figure 2C**). Similarly, 90% of C2 was in G3, significantly higher than 45% in C1 and 41% in C3 (**Figure 2D**). At the molecular subtype level, IDHwt, IDHmut-non-codel, and IDHmut-codel subtypes were mainly concentrated in samples with C2, C1, and C3 (**Figure 2E**). It is well known that IDH mutation and 1p19q co-deletion are favorable factors for the prognosis of gliomas. Also, WHO 2021 indicates that the IDHwt corresponds to the new WHO grade 4, IDHmut-non-codel corresponds to oligodendroglioma, and IDHmut-codel corresponds to oligodendroglioma (25). The prognosis of the latter of these three molecular subtypes is sequentially better than that of the former (1). In fact, in terms of histological distribution, the C2 subtype has the highest percentage of astrocytic origin, while the C3 subtype has the highest percentage of oligodendroglioma (**Supplementary Figure S2A**). Furthermore, among the supervised DNA methylation clusters, the codel and G-CIMP high subtypes were mainly concentrated in C3 and C1, whereas the G-CIMP low and the mesenchymal-like subtypes were almost exclusively present in C2 (**Figure 2F**). The original study showed that the codel subtype had the best prognosis, followed by the G-CIMP high subtype. However, the G-CIMP low subtype is more inclined to be observed in GBM, and the mesenchymal-like subtype is associated with high invasiveness (13). Both of these two subtypes generally have the worst prognosis.

Secondly, some specific gene copy number variation (CNV) events have also been associated with poor prognosis in gliomas. We calculated the probability of occurrence of seven CNV events, including CDKN2A/B deletion, chr7 gain & chr10 loss, chr19/20 co-gain, EGFR amplification, and PDGFRA/B deletion. We note that the proportion of these CNV events was much higher in C2 than it was in C1 and C3 (**Supplementary Figures S2B–H**). Evidence suggests that CDKN2A/B deletion is one of the characteristics of astrocytomas and that chr7 gain & chr10 loss and EGFR amplification are characteristics of glioblastomas (25). Thus, these results were consistent with the histological and molecular subtype characteristics observed above. In summary, abnormal glycolytic metabolism was often accompanied by changes in molecular profile. The unbalanced accumulation of multiple risk factors eventually led to the heterogeneity of prognosis among different glycolysis-related subtypes.

### 3.3 The immune landscape of the glycolysis-related subtypes

Previous studies have shown that abnormalities in glycolytic metabolism are associated with the generation of tumor immune escape (26). To verify this in LGG samples, we further depicted the immune landscape of different glycolysis-related subtypes.

**Figure 3:**
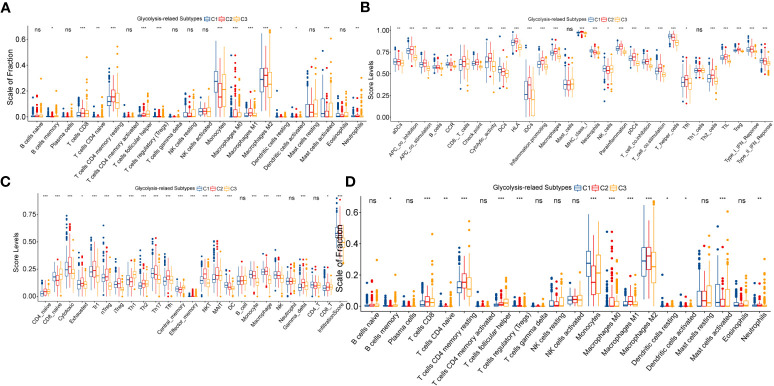
Variation in immune cell infiltration between three glycolysis-related subtypes evaluated by the CIBERSORT **(A)** and ImmuCellAI **(B)** algorithms. Variation in ssGSEA score levels of immune signatures between three glycolysis-related subtypes **(C)**. Differences in the activity of seven steps in the immune cycle between three glycolysis-related subtypes **(D)**. In this figure, variance analysis was performed with the K-S test. p<0.05 was presented with “*”, p<0.05 was presented with “**”, p<0.05 was presented with “***”. ns, not statistically significant.

Firstly, the ESTIMATE score increased sequentially among C3, C1, and C2 (**Supplementary Figure S3A**). This implied that the degree of immunosuppression and the complexity of the microenvironment might be sequentially increased. Next, we found that typical pro-tumor cells, such as M2 macrophages, memory CD4 T cells resting, and mast cell resting were significantly highly infiltrated in C2 (**Figure 3A**).

Interestingly, the proportion of anti-tumor effector cells, such as CD8 T cells and NK cells, was also highest in C2. Similarly, multiple pro- and anti-tumor immune signatures were simultaneously exhibited with the highest enrichment levels in C2, followed by C1, and the lowest in C3 (**Figure 3B**). Also, with the ImmuCellAI algorithm, we noted that not only anti-tumor signatures such as MAIT and Tfh, but also pro-tumor signatures like Th2, nTreg, and iTreg were most highly activated in the C2 (**Figure 3C**). Notably, C2 has the highest exhaustion signature. We speculated that T-cell exhaustion might be involved in the formation of this unique immune cell infiltration pattern. To explain this result, we further explored the differences in cancer immune cycles between samples with the TIP algorithm. As shown in **Figure 3D**, the cancer immune cycle in C2 is limited mainly by two processes: infiltration of T cells into the tumor and killing of cancer cells. In addition, the C2 subgroup had the highest immune dysfunction and exclusion levels in the TIDE algorithm-based assessment. The exclusion level in C1 was not remarkably distinct from that in C3, but its dysfunction level was much higher than that in C3 (**Supplementary Figures S3B, C**). Also, the expression of immune checkpoints, including CTLA4, PD-1, PD-L1, and PD-L2, increased sequentially among C3, C1, and C2 (**Supplementary Figure S3D**). ICPs are closely related to T-cell exhaustion. For example, PD-1 and its ligands PD-L1 & PD-L2 can suppress the function of effector T cells, and CTLA4 can bind CD80 and CD86 molecules to transduce T cell suppressive signatures and enhance the immunosuppressive ability of Tregs. Finally, we explored the distribution status of TCGA immune subtypes between the glycolysis-related subtypes. Immunologically quiet (IC5) and lymphocyte depleted (IC4) were dominant in LGG samples, while IC4 was predominantly distributed in the C2 subtype. Also, the proportion of IC4 was 22% in C1, which was significantly higher than that of 11% in C3 (**Supplementary Figure S3E**). The original study has illustrated that the immunological profile of IC4 is more confounded and has a worse prognosis compared to IC5 (14). The T-cell exhaustion level is higher in IC4 compared to IC5, which reasonably supports our results (14). In summary, the anti-tumor immunity decreased sequentially between C3, C1, and C2. Abnormalities in glycolytic metabolism might be associated with alterations in the immune regulation process of LGG, which finally manifested as diverse clinical outcomes.

### 3.4 Correlation of glycolysis-related subtypes with stemness, EMT, and energy metabolic features

In addition to immunological alterations, other microenvironmental features such as stemness and EMT processes are strongly associated with oncogenesis, progression, invasion, metastasis, and poor prognosis of cancers. Also, abnormal glycolytic metabolism is essentially a consequence of cancer cells responding to impaired energy metabolism. Therefore, we speculated that the heterogeneity in prognosis among different subtypes might be equally related to the alteration of these malignant features.

**Figure 4:**
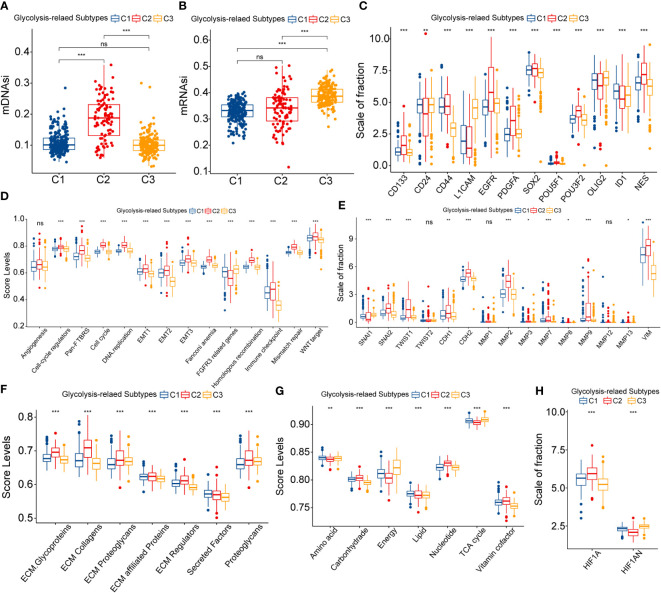
Differences in mDNAsi **(A)** and mRNAsi **(B)** between three glycolysis-related subtypes. Differential expression of glioma stem cell markers between three glycolysis-related subtypes **(C)**. Variation in ssGSEA score levels of hallmark cancer signaling pathways between three glycolysis-related subtypes **(D)**. Variation in the expression of epithelial-mesenchymal translation (EMT) central genes between three glycolysis-related subtypes **(E)**. Variation in the ssGSEA score levels of extracellular matrix (ECM) organization-related signaling pathways between three glycolysis-related subtypes **(F)**. Differential expression of HIF1A and HIF1AN between three glycolysis-related subtypes **(G)**. Differential expression of HIF1A andHIF1AN between three glycolysis-related subtypes **(H)**. In this figure, variance analysis was performed with the K-S test. p<0.05 was presented with “*”, p<0.01 was presented with “**”, p<0.001was presented with “***”. ns, not statistically significant..

Firstly, we evaluated the differences in stemness features between glycolysis-related subtypes. Based on OCLR machine learning, two stemness indices, methylated DNA-based stemness index (mDNAsi), and mRNA expression-based stemness index (mRNAsi), were obtained (27). We noted that C2 had the highest mDNAsi levels, whereas C3 had the highest mRNAsi levels (**Figure 4A**). mRNAsi did not significantly vary between C1 and C2, whereas C1 and C3 had no significant difference in mDNAsi levels (**Figure 4B**). In the context of glioma, mDNAsi was negatively correlated with prognosis, whereas mRNAsi was positively correlated with prognosis (27). Therefore, altered stemness features might be related to abnormal glycolytic metabolism and affect the prognosis of samples with different subtypes. Furthermore, among the glioma stem cell markers, C2 had the highest overall expression level, followed by C1, and the lowest in C3 (**Figure 4C**). These results suggested that the stemness levels might decrease sequentially between C2, C1, and C3.

The EMT process is often accompanied by changes in the stemness feature of cancers and is closely correlated with the invasion and metastasis of cancer. EMT-related signaling pathways (Pan-F-TBRS and WNT) and three groups of EMT level signatures were most enriched in C2 subtypes and least enriched in C3 (**Figure 4D**). Also, EMT transcription factors were highly expressed in C2 and presented the most pronounced inversion of CDH1/2 expression (**Figure 4E**). In addition, MMPs and VIM, which were closely associated with extracellular matrix remodeling, were equally highly expressed in C2 subtypes (**Figure 4E**). Also, ECM, cell cycle, and angiogenic signaling-related pathways were highly enriched in the C2 subtype (**Figures 4D, F**). In summary, EMT processes might be most active in C2 and could be widely involved in the development of poor prognosis in samples with C2.

Finally, we discussed the abnormalities of substance and energy metabolism among glycolysis-related subtypes. **Figure 4G** presented the activity of metabolism-related molecular functions between these three subtypes. Similarly, hyper-metabolism of nucleotides might be associated with the hyper-proliferation of cells. In contrast, the score levels of tricarboxylic acid (TCA) cycle and energy metabolism were the lowest in C2, which was disproportionate to the hyper-metabolism of carbohydrates. The rapid proliferation of tumor cells can lead to an increased degree of hypoxia, which in turn manifests as the activation of aerobic glycolysis and decreased glucose utilization and ATP generation rates (28). The expression of HIF1A decreased sequentially in C2, C1, and C3, and the expression of its repressor HIF1AN sequentially increased (**Figure 4H**). Above all, increased stemness and EMT levels contributed to the highly active invasion and proliferation of LGG cells. This could lead to increased hypoxia and restricted energy metabolism within the tumor, which eventually exhibited the generation and activation of aerobic glycolysis.

### 3.5 Genetic heterogeneity among glycolysis-related subtypes

Alteration of genetic characteristics is one of the hallmarks of cancer (29). To elucidate whether abnormal glycolytic metabolism was related to the genetic heterogeneity of LGG, we next depicted mutational profiles.

**Figure 5:**
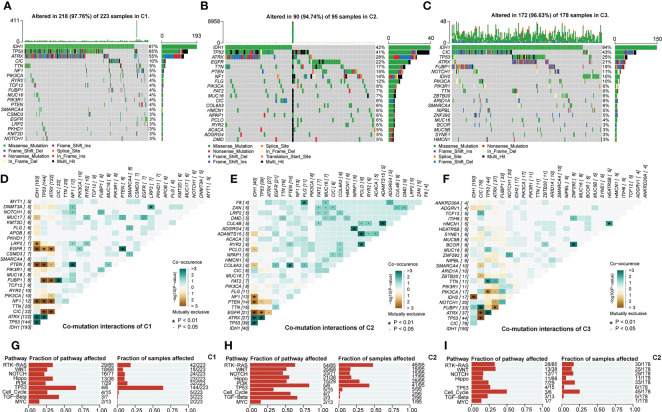
Overview of mutation rates and sites of 20 top mutated genes in C1 **(A)**, C2 **(B)**, and C3 **(C)**. Interaction of 30 top mutated genes in C1 **(D)**, C2 **(E)**, and C3 **(F)**. Functional annotation of the mutation profile in C1 **(G)**, C2 **(H)**, and C3 **(I)**. In this figure, the interaction of mutated genes was evaluated with Fisher’s exact test. p<0.05 was presented with “*”, p<0.05 was presented with “**”, p<0.05 was presented with “***”.

We noted that the mutation rates of ATRX and TP53 were significantly higher in C1 than in the other two subtypes, while the IDH1 mutation rate was not significantly different from C3 (**Figures 5A–C**). Evidence suggested that the IDH1 could co-mutate with ATRX and TP53 in gliomas. In our study, **Figures 5D–F** was consistent with this point of view. However, there is no absolute necessary correlation for this co-mutation relationship. Thus, the higher TP53 mutation rate in the C1 subtype may still have an adverse prognostic impact, manifesting as a worse prognosis in C1 than in C3. In addition, among the top 4 genes, there were large differences in the type, site, and frequency of mutations among the different subtypes except for IDH1 (**Supplementary Figures S4A–D**). This might be attributed to the conservative mutation type and site of IDH1. Also, these results implicated that distinct genetic heterogeneity existed among different subtypes, which might have an impact on the biological properties of LGG. **Figures 5G–I** presented the results of the functional enrichment analysis based on mutational profiles. The enrichment levels of these cancer signaling pathways were highest in C2, followed by C1, and lowest in C3. Among them, the WNT, NOTCH, PI3K, and TGF-β signaling pathways are closely related to EMT and cancer stem cell generation, and the RTK-RAS and PI3K signaling pathways can regulate cancer progression (30–33). In addition, the Hippo and WNT signaling pathways are associated with the acquisition of stem cell features, and the activation of the NOTCH signaling pathway can contribute to the formation of an immunosuppressive microenvironment (34–36). In addition, the PI3K/AKT pathway can promote the establishment of aerobic glycolysis through mTOR/HIF1α signaling, which performs a variety of biological functions (37). Therefore, cancer signaling pathways might serve as a bridge between the malignant features and glycolytic metabolic processes of LGG. Finally, we noted that oncogenes in C1 and C3 were mainly manifested as mutually exclusive mutations, while C2 was dominated by co-occurrence mutations (**Figures 5D–F**). his indicated that C2 might have higher genomic instability levels. The C2 subgroup had a median somatic mutation count of 48, much higher than 28 and 25 in C1 and C3 (**Supplementary Figures S4E**). Elevated genomic instability is a hallmark of poor prognosis in gliomas. In summary, variations in cancer signaling pathway activity accompanied by genetic heterogeneity might be closely related to the different biological features among glycolysis-related subtypes. The abnormal glycolytic metabolism levels were positively correlated with the malignancy of LGG.

### 3.6 Construction of the GlySig risk models

**Figure 6:**
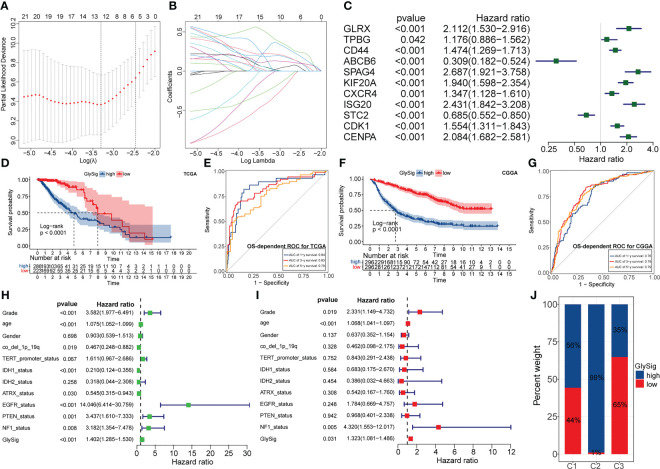
The relationship between penalty coefficient (log(λ)) values and partial likelihood deviance. The point with the lowest partial likelihood of deviance corresponds to the best log(λ) value **(A)**. Relationship between the weight coefficients (Coef) corresponding to the GlySig in the LASSO regression and log(λ) value **(B)**. Univariate-Cox regression analysis of lasso genes in the TCGA LGG cohort **(C)**. OS-dependent K-M curves between the high and low GlySig subtypes in the TCGA cohort **(D)**. 1-, 3- and 5-year OS-dependent ROC curves for the GlySig in the TCGA cohort **(E)**. OS-dependent K-M curves between the high and low GlySig subtypes in the CGGA cohort **(F)**. 1-, 3- and 5-year OS-dependent ROC curves for the GlySig in the TCGA cohort **(G)**. Univariate **(H)** and multivariate **(I)** Cox analysis of GlySig with clinical and molecular traits. Differential distribution of GlySig-related subgroups between three glycolysis-related subtypes **(J)**.

To quantify the abnormal glycolytic metabolism level and its prognostic impact, we constructed a prognostic model based on LASSO regression and the expression of 26 key genes. In the TCGA cohort, LGG samples were equally divided into the train (n = 256) and test (n = 253) sets. The chi-square test demonstrated that there was no significant bias in clinical traits between the train and test sets (**Supplementary Table 1**). After evaluation, the optimal penalty coefficient (log(λ)) was -3.3, at which point 11 genes had non-zero Coef values (**Figures 6A, B**). Furthermore, all these 11 genes had significant prognostic significance in LGG (**Figure 6C**). Therefore, these 11 genes were finally identified as lasso genes for the construction of GlySig (**Supplementary Table 2**).

Next, we validated the prognostic significance of GlySig. The K-M curves showed that the high GlySig subgroup had a worse prognosis (**Figure 6D**). Meanwhile, the AUC values of GlySig predicting 1-, 3-, and 5-year OS were more stable in the TCGA cohort, at 0.84, 0.83, and 0.78 (**Figure 6E**). Similarly, GlySig was also a risk factor for prognosis in the CGGA cohort, and its corresponding AUC values for 1-, 3-, and 5-year OS were 0.76,0.79, and 0.79 (**Figures 6F, G**). Thus, GlySig maintained a stable and accurate prognostic predictive capability across different databases, patient sources, and ethnographic conditions. In addition, GlySig was a significant prognostic risk factor in both univariate and multivariate Cox analyses (**Figures 6H, I**). Also, the prognosis of the low GlySig subgroup was significantly better than that of the high GlySig subgroup in all three-sample stratification based on the new WHO 2021 glioma grades (**Supplementary Figures S4F–H**). In summary, GlySig could be competent as an independent prognostic biomarker for LGG.

**Figure 7:**
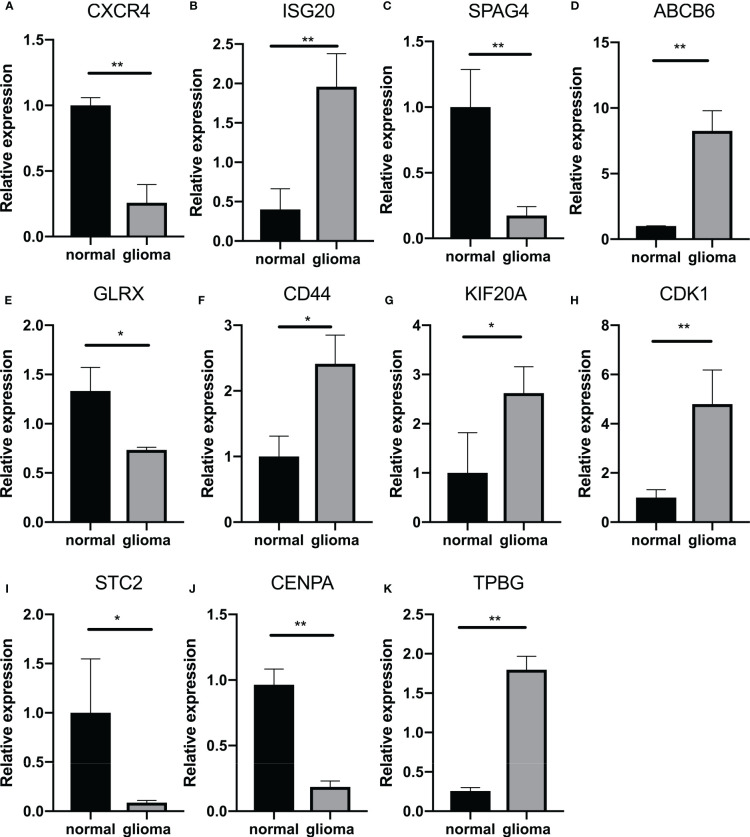
Quantitative analysis of GlySig-related genes using qRT-PCR: **(A–K)**. Relative differences in gene expression of GlySig-related genes between normal and astrocytic glioma cell lines. In this figure, p<0.05 was presented with “*”, p<0.01 was presented with “**”, p<0.001 was presented with “***”.

Finally, we attempted to identify the relationship between GlySig and glycolysis-related subtypes. As shown in **Supplementary Figures S4I**, GlySig was increased sequentially among C3, C1, and C2. Also, we noted that 99% of samples in C2 were simultaneously located in the high GlySig subgroup (**Figure 6J**). These results suggested that the biological properties of the high GlySig subgroup might be more inclined to samples in C2. In addition, glycolysis-related pathways such as aerobic glycolysis and Feeder pathways were highly enriched in high GlySig, while electron transport, tricarboxylic acid (TCA) cycle and gluconeogenic signatures were relatively underactive in the high GlySig subgroup (**Supplementary Figures S4J**). In addition, we verified the expression of lasso gene by PCR experiments (**Figures 7A–K**) This implied that GlySig was positively correlated with glycolytic metabolic activity. Above all, GlySig was a reliable biomarker that could independently predict prognosis and reflect the glycolytic metabolic activity in LGG, which had potential clinical applicability.

### 3.7 Co-expression and functional annotation of LASSO genes

To further reveal the functions of lasso genes and their mechanisms affecting the malignant progression of LGG, we further constructed the co-expression network and annotated their function. Firstly, a total of 878 DEGs were identified between the high and low GlySig subgroups with the |logFC|>1 and FDR<0.05 as thresholds (**Supplementary Figures S5A**). Next, by Spearman correlation analysis, we examined the correlations between the expression of lasso genes and DEGs. With a correlation coefficient (Cor) >0.5 and p<0.05 as thresholds, 485 interaction relationships were retained, which contained 217 nonreplicated DEGs (**Supplementary Figures S5B**). Finally, we performed the GO and KEGG functional enrichment analysis on the nodes of the coexpression network. The results indicated that these nodal genes were mainly enriched in pathways related to cell cycle regulation . These nodal genes regulated some immunoregulatory signaling pathways, such as the MHC II signaling pathway (**Supplementary Figures S5C, D**). Thus, apart from being a prognostic marker the lasso genes were also extensively involved in the malignant progression of LGG. These findings partially revealed the mechanism by which abnormal glycolytic metabolism affects the malignant features of LGG and further supported the reliability of GlySig as a biomarker.

### 3.8 The nomogram construction

**Figure 8:**
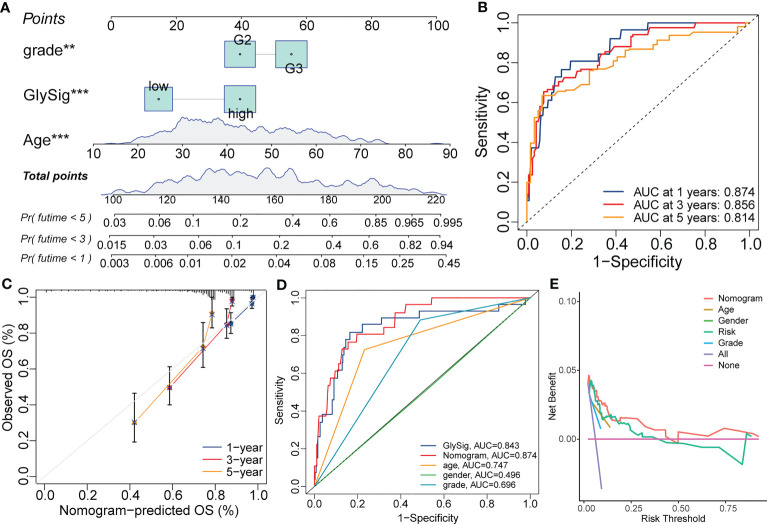
The nomogram constructed with the GlySig, grade, and age with the Cox regression for the prognostic prediction of LGG samples in the TCGA cohort **(A)**. 1-, 3-, and 5-year OS-dependent ROC curves for the nomogram **(B)**. Calibration curves for the 1-, 3-, and 5-year OS prediction of the nomogram **(C)**. Comparison of OS-dependent ROC curves for the nomogram with other single-trait prognostic prediction strategies **(D)**. Decision curves for the nomogram and other single-trait prognostic prediction strategies **(E)**. In this figure, p<0.01 was presented with “**”, and p<0.00 1 was presented with “***”.

The results of multivariate Cox analysis indicated that GlySig was a prognostic risk factor independent from clinical and molecular traits (**Figure 6I**). To achieve higher clinical benefits for LGG patients, we constructed the nomogram by integrating molecular profiles and GlySig (**Figure 8A**). The ROC curves showed that the AUC values for the 1-,3-, and 5-year OS prediction by the nomogram were 0.874, 0.856, and 0.814 (**Figure 8B**). Meanwhile, the calibration curve revealed that the error in the prognostic prediction of the nomogram was within an acceptable range (**Figure 8C**). These results indicated that the prognostic predictive capability of the nomogram was reliable. In addition, in the OS-dependent ROC curves, the nomogram had the highest AUC value (**Figure 8D**). Finally, we performed the decision curve analysis (DCA). The DCA curves showed that the nomogram had the highest net clinical benefit compared to a single-indicator prognostic evaluation strategy (**Figure 8E**). In summary, the prognostic predictive capability of the nomogram in LGG was more accurate than GlySig. At the same time, nomograms could bring higher net clinical benefits and have broader clinical applications. The analysis and design flow chart of this article is placed in the **Supplementary Figures S6**.

## 4 Discussion

Our findings demonstrated the metabolic heterogeneity of lower-grade gliomas and describe the clinical significance, molecular differences and immunological characteristics of three different glycolytic subtypes. The three different glycolytic subtypes, named C1, C2 and C3, were identified, with C3 having the best prognosis, followed by C1, and C2 having the worst prognosis. They corresponded to IDHmut-non-model, IDHwt and DHmut-model, respectively. where the C2 type showed distinct T-cell failure features. In addition, we developed GlySig to reflect the glucose metabolic activity of LGG, suggesting that high levels of our glucose metabolism are closely associated with poor prognosis. Our findings explained and characterized the heterogeneity of glucose metabolism within diffuse LGG and provide new ideas for the treatment of glioma.

We noted extensive molecular profiling heterogeneity among the three glycolysis-related subtypes, with C1, C2, C3 subtypes enriched in IDHmut-non-codel, IDHwt, and IDHmut-codel subtypes, respectively. In the DNA methylation cluster, G-CIMP low and mesenchymal-like subtypes are almost exclusively present in C2, where C1 and C3 are enriched in G-CIMP high and codel subtypes, respectively. With the analysis of cancer metabolism and genetics over the past two decades, IDH1 mutations not only lead to the accumulation of 2-hydroxyglutaric acid, but also to extensive changes in metabolic strategies, and metabolic reprogramming can be largely seen as a result of oncogenic driving events (38). IDH mutations not only affect amino acid metabolism but also lower glucose oxidation through inhibitory phosphorylation of pyruvate dehydrogenase (PDH). In addition, IDH1 mutant glioma cells show greater flux through pyruvate carboxylase leading to greater production of oxaloacetate. These results suggest that IDH1 mutant glioma cells adaptively run the Kreb’s Cycle backwards, perhaps to produce sufficient succinate to power the electron transport chain (38–40). In contrast, for the IDHwt mesenchymal subtype glioma promoter region is hypermethylated, resulting in increased glycolytic enzyme gene expression (41). Consistent with our results, the IDHwt type accounted for the highest C2 subtype with higher levels of glycolysis.

Studies have shown that exposure to different oxygen gradients leads to different metabolism in glioma cells. The transcription factor HIF-1a is activated and stabilized under hypoxic conditions, leading to a shift towards glycolysis and angiogenesis (42, 43). The Warburg effect is a key contributor to the adaptation of tumor cells to metabolic stress (5). Our results show that the expression of HIF1A decreases sequentially in C2, C1 and C3, while the expression of its suppressor HIF1AN increases sequentially.In addition, increased hypoxia and aerobic glycolysis ultimately lead to increased lactate production. HIF-1a activates aldolase, glyceraldehyde-3-phosphate dehydrogenase, lactate dehydrogenase, membrane lactate transporter protein (MCT4), and carbonic anhydrase 9 and 12, all of which stimulate glycolytic fluxes and facilitate lactate shuttling to the extracellular space (5). Lactate can be used to acidify the microenvironment and promote invasion (28). Tumor acidification has been shown to promote the expression of GSC markers and self-renewal of GSC in gliomas, and the GSC itself promotes the paracrine loop, thereby facilitating further expression of HIF1/2a in the stem compartment. *In vitro* studies have also shown that modulating pH can affect HIF2a expression and subsequently reduce GSC self-renewal capacity, suggesting that keeping lower PH is beneficial for tumors to maintain the depots of their GSCs and ultimately maintain tumor growth and resistance (44). Consistent with our findings, hypoxia levels in different glycolytic subtypes were consistent with stemness levels, with the C2 subtype having the highest stemness levels, EMT levels and high expression of HIF-1a, contributing to the highly active invasive behavior of LGG cells.

Aerobic glycolysis in tumors is an important cause of the immunosuppressive microenvironment in tumors. Rapid glucose utilization and increasing oxygen demand in tumors lead to hypoxia and induction of hypoxia-inducible factor 1α, which increases transforming growth factor (TGF)-β production, thereby further suppressing NK cells and stimulating immunosuppressive CD4+ T cells (6), In addition, lactate accumulation due to aerobic glycolysis also has direct immunosuppressive effects at many levels, including inhibition of monocyte differentiation to DCs and suppression of T cell responses (7, 8). Among our results, it is noteworthy that the C2 subtype has the highest depletion profile. the cancer immune cycle in C2 is limited by two main processes: T cell infiltration into the tumor and killing of cancer cells, and we speculate that T cell depletion may be involved in the formation of this unique pattern of immune cell infiltration. It has been shown that the need for effective T cell activation is met by upregulation of metabolism (45, 46). While HGG aerobic glycolysis is increased, tumor cells compete with effector T cells for glucose in TME. For example, the proliferation of CD4+ T cells and CD8+ T cells requires the glycolytic intermediate 2-phosphoenolpyruvate (PEP), which is essential for nuclear translocation in response to T cell receptor stimulation (47, 48). Similarly, glycolysis itself can control the function of effector T cells by inhibiting the RNA binding of glycolytic enzymes such as GAPDH and LDH and preventing their efficient translation. Combined with our results we suggest that the C2 subgroup has the highest level of T cell failure and immune dysfunction (**Supplementary Figure S3B, C**).

There are still some limitations in this study. First, we used a functional classification score (FCS) approach to quantify phenotypes and pathways, but FCS analyzes each pathway independently, and since the same gene may be involved in multiple pathways, this may lead to significant enrichment of individual pathways due to gene overlap.In addition, treating each gene as an individual ignores the biological properties of genes and the complex interactions between genes. Second, although the correlation between metabolic levels and infiltrating immune cells was determined at the macroscopic level, the underlying mechanisms of metabolism of these immune cells are unknown, Experimental studies should be conducted on the metabolism of immune cells to explore their functional roles in greater depth.

## 5 Conclusion

This study includes a multi-omics analysis of the glycolysis-associated gene set to explore the relevance of glycolysis to lower-grade glioma heterogeneity and to develop a glycolysis risk scoring system. We retrieved data from TCGA including mutations, DNA methylation, mRNA expression, histopathological grading, molecular subtypes and clinical parameters. Unsupervised clustering analysis of core glycolytic genes identified three different glycolytic subtypes, named C1, C2 and C3, with C3 having the best prognosis, followed by C1, and C2 having the worst prognosis. The clinical features, immune infiltration, metabolic features and somatic cell variants were also explored. In order to quantify the level of abnormal glucose metabolism and its impact on prognosis, we developed GlySig to reflect the glucose metabolic activity of LGG and integrated molecular features to construct a nomogram that can be independently assessed to predict prognosis. Our results explain and characterize the heterogeneity of glucose metabolism within diffuse LGGs, providing new ideas for the treatment of glioma and expanding our understanding of glioma heterogeneity.

## Data availability statement

The original contributions presented in the study are included in the article/**Supplementary Material**. Further inquiries can be directed to the corresponding authors.

## Author contributions

BL, SW conceived and designed the study. LS, SF and XG were responsible for data collection, analysis, and checking. SW wrote the original draft of the manuscript, which was reviewed and revised by CW. BZ and BL supervised the study. All authors contributed to the article and approved the submitted version.

## Supplementary material

Supplementary Figure 1 Five glycolysis-related signaling pathways that were highly enriched in LGG samples compared with normal cortical samples in GTEx, including GOBP_FRUCTOSE_1_6_BISPHOSPHATE_METABOLIC_PROCESS (A); REGULATION_OF GLYCOLYSIS_BY_FRUCTOSE_2_6_BISPHOSPHATE_METABOLISM (B); GLYCOLYSIS IN SENESCENCE (C); HALLMARK_GLYCOLYSIS (D); and REACTOME_GLYCOLYSIS (E). Differences in the distribution of principal components among glycolysis-related subtypes. Points with varied colors represented samples in different subgroups.

Supplementary Figure 2 Differential distribution of Histology **(A)**, chr7 gain & chr10 loss **(B)**; CDKN2B status **(C)**, CDKN2A status **(D)**, EGFR status **(E)**; chr19 and chr20 co-gain **(F)**; PDGF2A status **(G)**; and PDGF2B status **(H)** between three glycolysis related-subtypes.

Supplementary Figure 3 Differences in ESTIMATE score **(A)**, Dysfunction levels **(B)**; and Exclusion levels **(C)** between three glycolysis-related subtypes. Differential expression of immune checkpoints between three glycolysis-related subtypes **(D)**. Differential distribution of TCGA immune subtypes between three glycolysis-related subtypes **(E)**. In this figure, variance analysis was performed with the K-S test. p<0.05 was presented with “*”, p<0.05 was presented with “**”, p<0.05 was presented with “***”.

Supplementary Figure 4 The lollipop plot demonstrated the differences in mutation rates and sites between different glycolysis-related subtypes for ATRX **(A)**; TP53 **(B)**; EGFR **(C)**; and IDH1 **(D)**. Differences in somatic mutation counts between three glycolysis-related subtypes **(E)**. OS-dependent survival curves between high and low GlySig subgroups in three WHO2021 stratifications, including IDHwt **(F)**, IDHmut-non-codel **(G)**, and IDHmut-codel **(H)**. Differences in GlySig **(I)** and glycolysis-related pathways **(J)** between three glycolysis-related subtypes.

Supplementary Figure 5 Volcano plot presented DEGs between the high and low GlySig-related subgroups **(A)**. Red points represented the DEGs highly expressed in the high GlySig subgroup, while the blue points represented the DEGs lowly expressed in the high GlySig subgroups. Black points mean DEGs did not fulfill the threshold of |logFC|>1 and FDR<0.05. Co-expression network of lasso genes with selected DEGs **(B)**. GO **(C)** and KEGG **(D)** functional enrichment analysis of nodes in the co-expression network.

Supplementary Figure 6 Article Analysis Design Flowchart.
