# Supplementary figures and images for: Heterogeneity in clinical prognosis, immune infiltration and molecular characteristics of three glycolytic subtypes in lower-grade gliomas
Source: Front Oncol. 2023 May 18;13:1180662. doi: 10.3389/fonc.2023.1180662 (PMC10233122; doi:10.3389/fonc.2023.1180662)

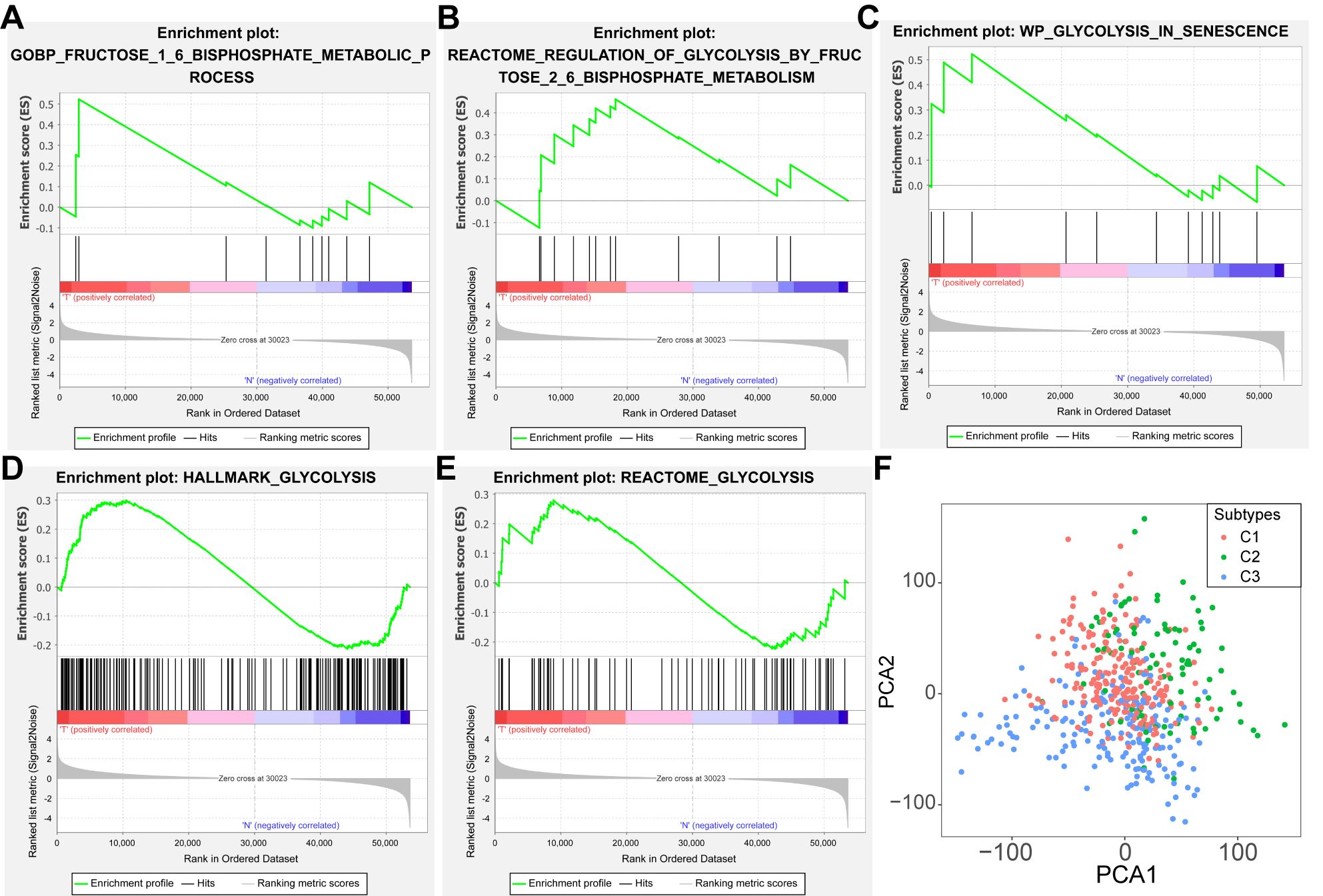

Supplement: Supplementary Figure 1 — Five glycolysis-related signaling pathways that were highly enriched in LGG samples compared with normal cortical samples in GTEx, including GOBP_FRUCTOSE_1_6_BISPHOSPHATE_METABOLIC_PROCESS (A); REGULATION_OF GLYCOLYSIS_BY_FRUCTOSE_2_6_BISPHOSPHATE_METABOLISM (B); GLYCOLYSIS IN SENESCENCE (C); HALLMARK_GLYCOLYSIS (D); and REACTOME_GLYCOLYSIS (E). Differences in the distribution of principal components among glycolysis-related subtypes. Points with varied colors represented samples in different subgroups. [file Image_1.tif]

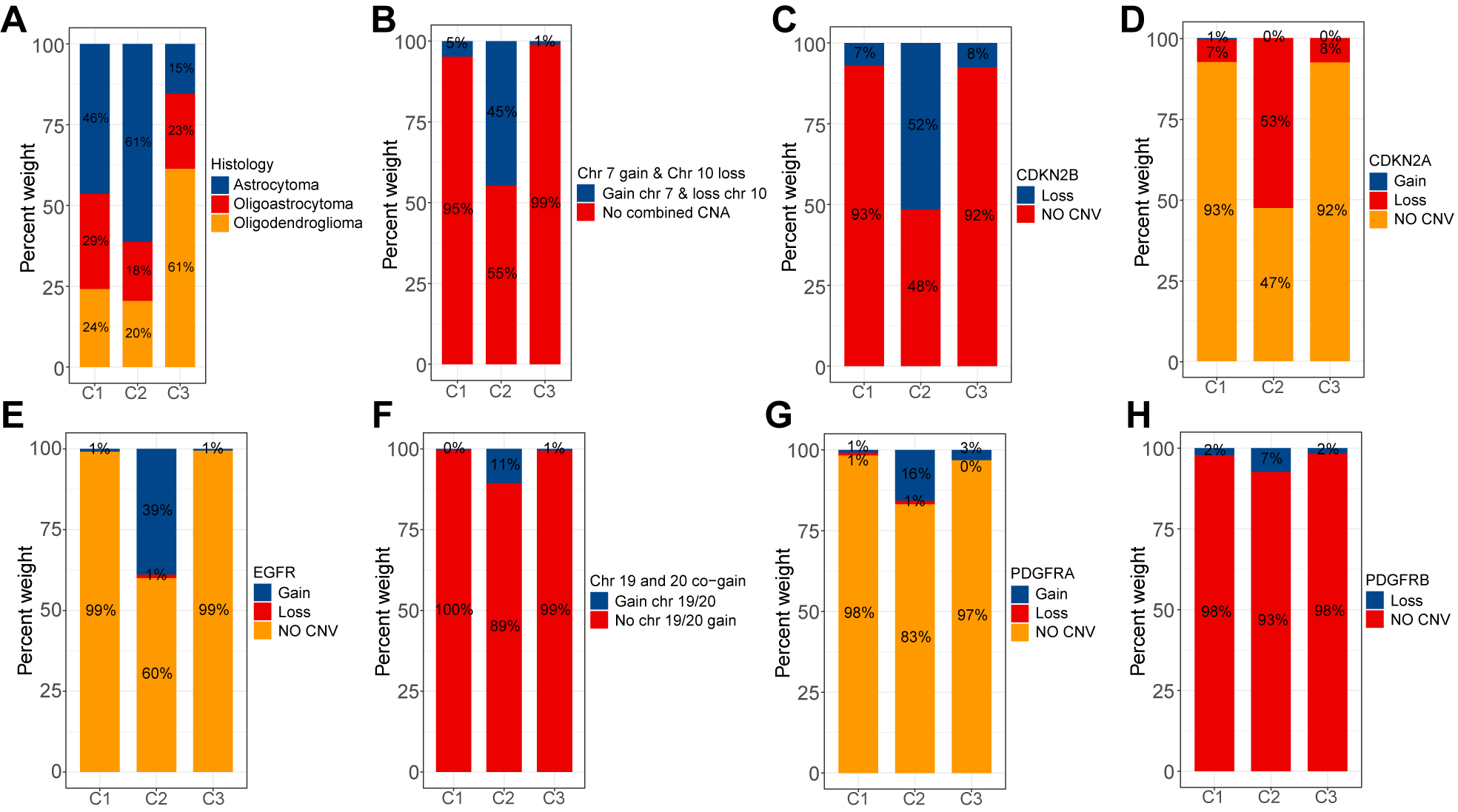

Supplement: Supplementary Figure 2 — Differential distribution of Histology (A), chr7 gain & chr10 loss (B); CDKN2B status (C), CDKN2A status (D), EGFR status (E); chr19 and chr20 co-gain (F); PDGF2A status (G); and PDGF2B status (H) between three glycolysis related-subtypes. [file Image_2.tif]

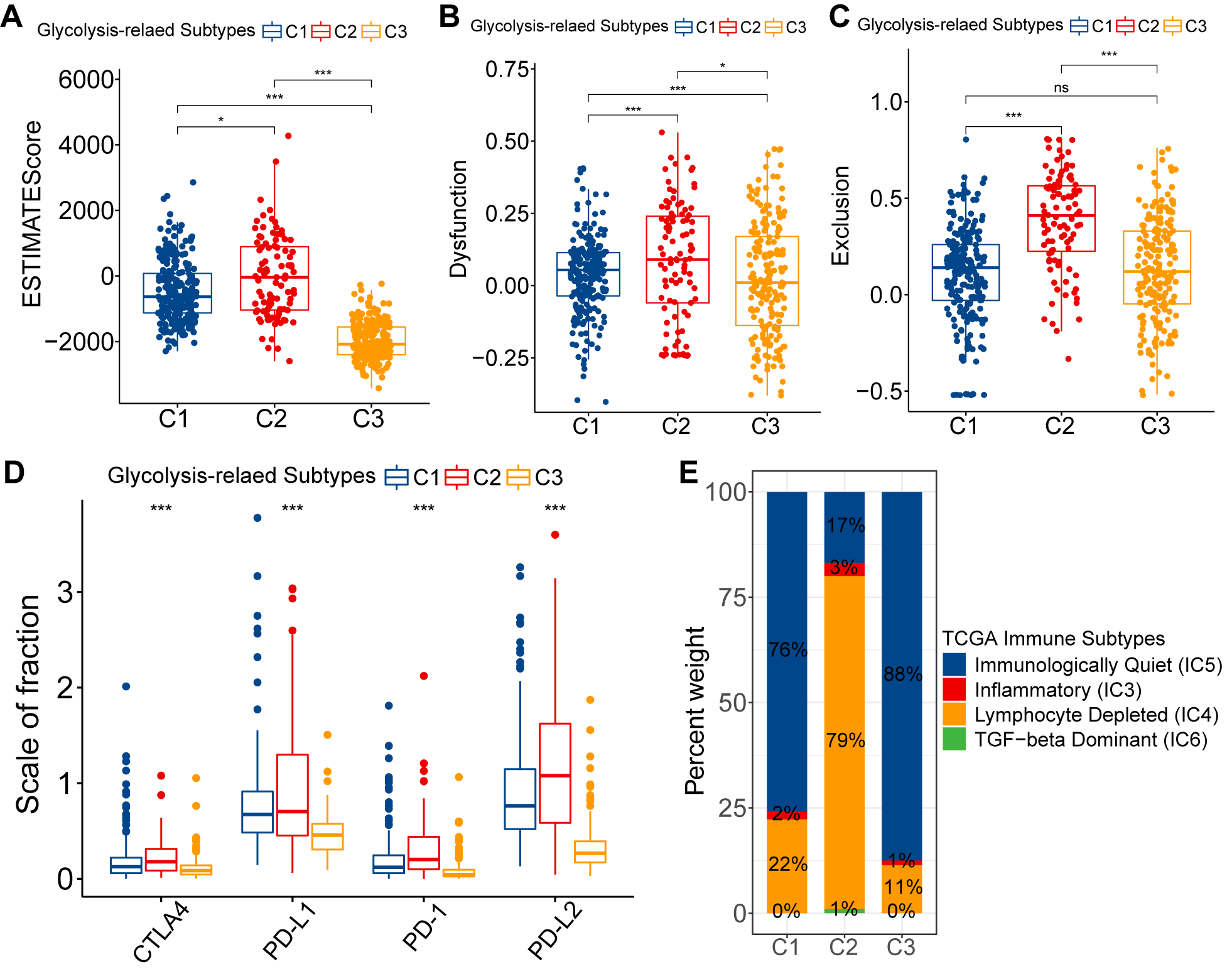

Supplement: Supplementary Figure 3 — Differences in ESTIMATE score (A), Dysfunction levels (B); and Exclusion levels (C) between three glycolysis-related subtypes. Differential expression of immune checkpoints between three glycolysis-related subtypes (D). Differential distribution of TCGA immune subtypes between three glycolysis-related subtypes (E). In this figure, variance analysis was performed with the K-S test. p<0.05 was presented with “*”, p<0.05 was presented with “**”, p<0.05 was presented with “***”. [file Image_3.tif]

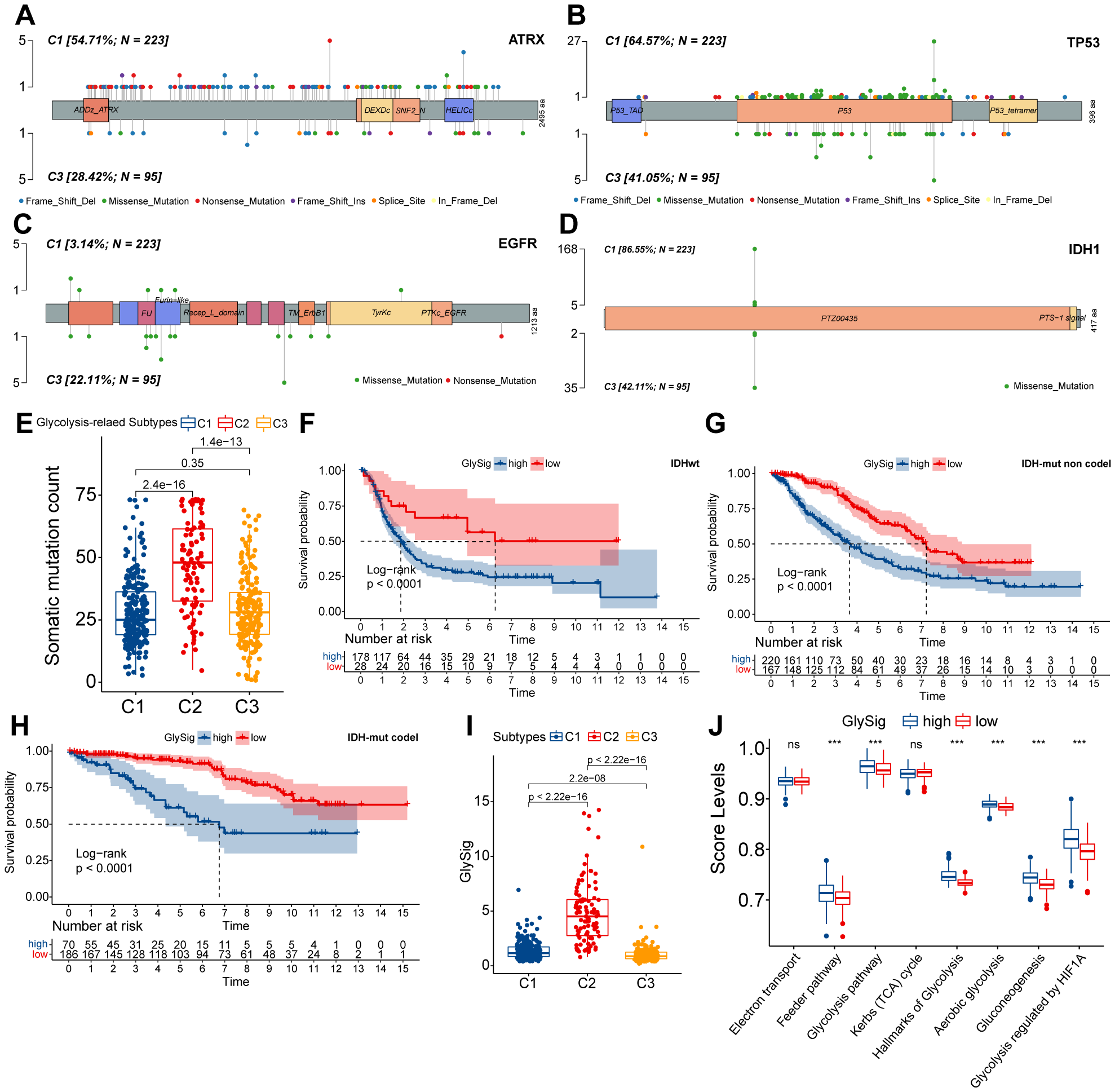

Supplement: Supplementary Figure 4 — The lollipop plot demonstrated the differences in mutation rates and sites between different glycolysis-related subtypes for ATRX (A); TP53 (B); EGFR (C); and IDH1 (D). Differences in somatic mutation counts between three glycolysis-related subtypes (E). OS-dependent survival curves between high and low GlySig subgroups in three WHO2021 stratifications, including IDHwt (F), IDHmut-non-codel (G), and IDHmut-codel (H). Differences in GlySig (I) and glycolysis-related pathways (J) between three glycolysis-related subtypes. [file Image_4.tif]

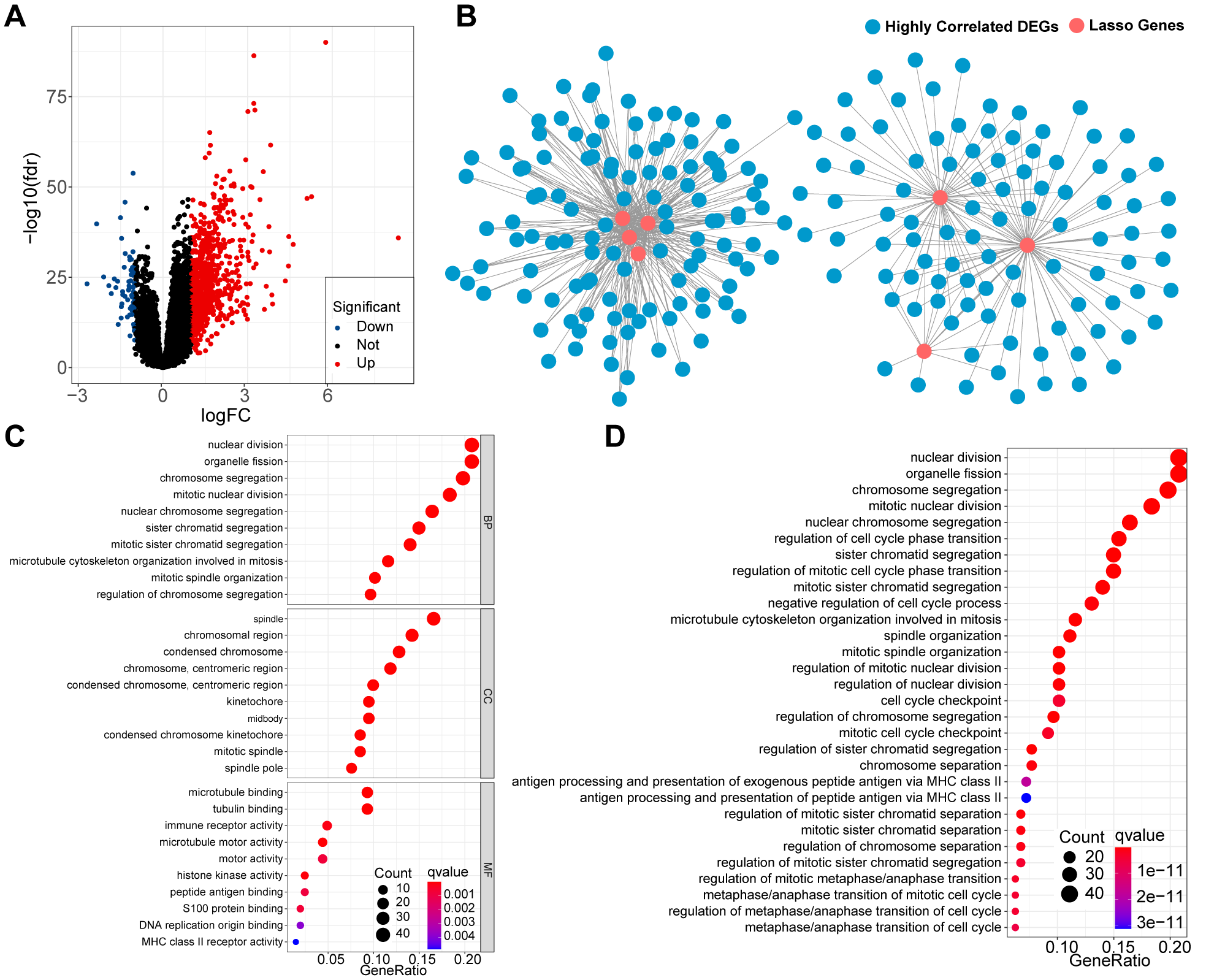

Supplement: Supplementary Figure 5 — Volcano plot presented DEGs between the high and low GlySig-related subgroups (A). Red points represented the DEGs highly expressed in the high GlySig subgroup, while the blue points represented the DEGs lowly expressed in the high GlySig subgroups. Black points mean DEGs did not fulfill the threshold of |logFC|>1 and FDR<0.05. Co-expression network of lasso genes with selected DEGs (B). GO (C) and KEGG (D) functional enrichment analysis of nodes in the co-expression network. [file Image_5.tif]

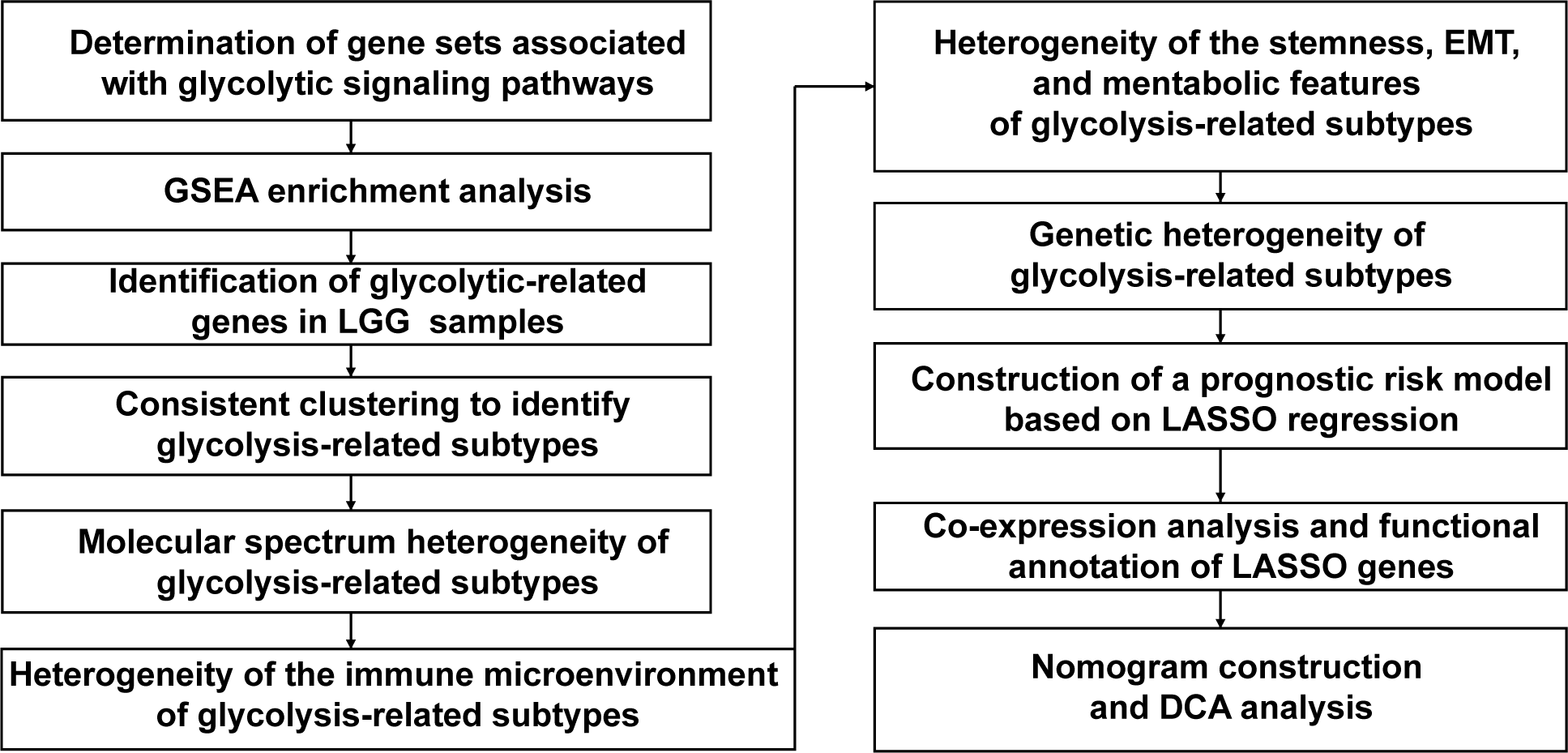

Supplement: Supplementary Figure 6 — Article Analysis Design Flowchart. [file Image_6.tif]
